# *CTLA4* Autoimmunity-Associated Genotype Contributes to Severe Pulmonary Tuberculosis in an African Population

**DOI:** 10.1371/journal.pone.0006307

**Published:** 2009-07-17

**Authors:** Thorsten Thye, Genevieve Scarisbrick, Edmund N. L. Browne, Margaret Amanua Chinbuah, John Gyapong, Ivy Osei, Ellis Owusu-Dabo, Stefan Niemann, Sabine Rüsch-Gerdes, Christian G. Meyer, Rolf D. Horstmann

**Affiliations:** 1 Department of Molecular Medicine, Bernhard Nocht Institute for Tropical Medicine, Hamburg, Germany; 2 Institute of Medical Biometry and Statistics, University Hospital Schleswig-Holstein, Campus Lübeck, Lübeck, Germany; 3 Department of Radiology, Komfo Anokye Teaching Hospital, Kumasi, Ghana; 4 Department of Community Health, College of Medical Sciences, Kwame Nkrumah University of Science and Technology, Kumasi, Ghana; 5 Health Research Unit, Ghana Health Service, Ministry of Health, Accra, Ghana; 6 Kumasi Centre for Collaborative Research in Tropical Medicine, Kumasi, Ghana; 7 National Reference Center for Mycobacteria, Research Center Borstel, Borstel, Germany; Institut de Pharmacologie et de Biologie Structurale, France

## Abstract

The gene of Cytotoxic T Lymphocyte-associated Antigen 4 (*CTLA4*), a negative regulator of T lymphocytes, contains a single-nucleotide polymorphism (SNP) at position +6230A->G (ct60A->G), which has been found associated with several autoimmune diseases and appears to reduce T-cell inhibitory activity. In Ghana, West Africa, we compared the frequencies of *CTLA4* +6230 A/G and 6 haplotype-tagging SNPs in 2010 smear-positive, HIV-negative patients with pulmonary tuberculosis (TB) and 2346 controls matched for age, gender and ethnicity. We found no difference in allele frequencies between cases and controls. However, +6230A and a distinct *CTLA4* haplotype and a diplotype comprising the +6230A allele were significantly less frequent among cases with large opacities in chest radiographs compared to those with small ones (P_corrected [cor]_ = 0.002, P_cor_ = 0.00045, P = 0.0005, respectively). This finding suggests that an increased T-cell activity associated with the *CTLA4* +6230G allele contributes to pathology rather than to protection in pulmonary TB.

## Introduction

Cytotoxic T Lymphocyte-associated Antigen 4 (CTLA-4) is considered a key negative regulator of T lymphocytes [1]. Being transiently surface expressed and released by activated T cells, it inhibits T-cell functions through a variety of direct and indirect mechanisms [2]–[7]. In addition, CTLA-4 has been found to skew the activity of T-helper (Th) cells towards a Th1 phenotype [8]–[9].

A G→A nucleotide exchange at position +6230 (also addressed as CT60; according to alternate numbering located at position +6253 relative to the start codon) in the 3′-untranslated region of the human CTLA-4 gene has been found to support protection from several autoimmune diseases [10]. It was associated with an increased concentration of a splice variant of the *CTLA4* transcript encoding a soluble form of the molecule, which was assumed to enhance the T-cell inhibitory function [10], [11]. The authors reasoned that the autoimmunity-supporting +6230G allele of *CTLA4*, which was found in the majority of members of the European study group, may have been the subject of evolutionary selection by providing enhanced cellular immune responses and, thereby, protection against particular intracellular infections [10]. The identification of this polymorphism provided an important tool to study the overall influence of T-cell responses in humans.

We studied the *CTLA4* +6230 polymorphism in pulmonary tuberculosis (TB) for two reasons. First, T cells are regarded of eminent importance in controlling mycobacterial infections [12]. In addition, TB may be relevant in the evolution of the autoimmunity-associated +6230G allele because it is caused by an intracellular infection and, due to its widespread occurrence and high mortality in the past and today, it may be relevant to natural selection.

Studying an African population, we included in the analysis four single nucleotide polymorphisms (SNP) which recently have been described to characterize the relevant haplotypes of the *CTLA4* gene in Africans [13] and, according to the most recent version of the HapMap database (http://www.hapmap.org/) five additional tagging SNPs providing comprehensive and appropriate coverage of the *CTLA4* gene.

## Materials and Methods

### Study group

Study participants were enrolled in Ghana, West Africa, between September 2001 and July 2004. The study group has been described in detail elsewhere [14]. In brief, patients were recruited at the two major teaching hospitals of Accra and Kumasi, and additional hospitals or policlinics in Accra, Kumasi, Obuasi, Agona, Mampong, Agogo, Konongo, Nkawie, Nkawkaw, Atibie, Assin Fosu and Dunkwa. The characterization of patients comprised a documentation of medical histories on standardized questionnaires, two examinations for acid-fast bacilli of non-induced sputum specimens, a determination of HIV serology and confirmation of positive results by an alternative test, culturing and differentiation of mycobacterial isolates by IS*6110* fingerprinting and spoligotyping as well as assessment of drug resistances, and a posterior-anterior chest radiography.

Included were patients 6 to 60 years of age, with no history of previous TB or anti-mycobacterial treatments, two sputum smears positive for acid-fast bacilli, and a negative HIV serology. Excluded were individuals with incomplete information provided by the questionnaire or evidence for alcoholism, drug addiction or other apparent generalized disease.

Unrelated personal contacts and neighbours as well as community members were recruited as controls. Inclusion criteria were no history of previous TB or anti-mycobacterial treatments and no evidence for previous TB in chest radiographs. The characterization of controls included a tuberculin skin test (Tuberculin Test PPD Mérieux, bioMérieux, Nürtingen, Germany).

Radiographic films were pseudonymized and read by an experienced radiologist (G. S.). Opacities, cavities, nodular lesions, pulmonary shrinkage (loss of lung volume as assessed by hilus dislocation or displacement of fissures), calcifications, and pleural thickening were individually assigned to the upper right, lower right, upper left, and lower left thoracic quadrants and overall assessed quantitatively being rated “0” (no lesion detectable), “1” (mild lesion), “2” (moderate lesion), and “3” (severe lesion). Enlargements of mediastinal lymph nodes, effusions, and miliary lesions were evaluated qualitatively. The evaluation presented here for the reason of simplicity was restricted to the overall scores for opacities and cavities because these are the most common signs of TB reflecting cellular infiltration and necrosis, respectively (Table 1).

**Table 1:** Radiographic findings in the case group^1^.

Severity score	Opacities	Cavities
0	17 (0.9%)	103 (5.6%)
1	340 (18.4%)	825 (44.6%)
2	1121 (60.6%)	682 (36.8%)
3	373 (20.2%)	241 (13.0%)

1 Shown are numbers of patients and, in brackets, percentages of total (n = 1851).

The final study group comprised 2010 cases and 2346 controls comprising 1211 household contacts and 1135 neighbours or community controls. They belonged to the following ethnic groups (cases/all controls/PPD-positive controls/PPD-negative controls): Akan including Ashanti, Fante, Akuapem (63.6%/59.1%/58.2%/74.8%), Ga-Adangbe (14.5%/19.8%/20.6%/7.0%), Ewe (7.1%/9.3%/9.6%/3.9%) and ethnic groups of northern Ghana, including Mole Dagbane, Gurma, Grusi (12.9%/10.4%/10.3%/12.6%). For 1.6% of study participants the ethnicity was not clearly assignable. The percentage of males among cases, all controls, PPD-positive controls and PPD-negative controls was 68.2%/59.2%/60.2%/42.5%, respectively. The mean age of study participants in these groups was 34.1±11.6/32.5±12.0/32.4±11.8/34.1±15.3.

The study protocol was approved by the Committee on Human Research, Publications and Ethics, School of Medical Sciences, Kwame Nkrumah University, Kumasi, and the Ethics Committee of the Ghana Health Service, Accra. Patients were treated according to the „Directly Observed Treatment, Short-course” (DOTS) strategy organized by the National Tuberculosis Programme. Blood samples for genetic analyses and HIV testing were taken only after a detailed explanation of the study aims and written or thumb-printed consent for participation provided, including HIV testing. Disclosure of HIV test results was dependent on the documented willingness of participants to be informed. HIV-positive patients were promptly referred to counseling and treatment as provided by the Ghanaian AIDS Control Programme.

### Genetic Analysis

After DNA extraction from whole peripheral blood by a magnetic separation technology (AGOWA®, Berlin, Germany), the *CTLA4* −1765 (rs11571315), −1722 (rs733618), −1661 (rs4553808), −1577 (rs11571316), −1411 (rs16840252), −443 (rs231774), +49 (rs231775), +923 (rs231777) and +6230 (rs3087243) variants were analysed by dynamic allele specific hybridization with fluorescence resonance energy transfer (FRET) in a LightTyper (Roche Diagnostics, Mannheim, Germany). Primer pair and sensor/anchor oligonucleotides for LightTyper-based genotyping are listed in Table 2.

**Table 2:** Primers and sensor/anchor oligonucleotides used for *CTLA4* genotyping^1^.

*CTLA4* SNP	rs Number	Primer Oligonucleotides^2^	Sensor/Anchor Oligonucleotides^3^
−1765 A/G	rs11571315	F–CTGCTAAGAGCATCCGC	S–TCAACTCCAGCATTGATCTCACTCTAT
		R–TGTTGGTGTGATGCACAG	A–GCTGAACCACTGGCTTCTGCTCCTCTACATAATAC
−1722 A/G	rs733618	F–CTGCTAAGAGCATCCGC	S–GGGTTTAGCTGCCTGTCCC
		R–TGTTGGTGTGATGCACAG	A-GCCACTGCTGTGTGTTCCTCTTGAGG
−1661 A/G	rs4553808	F–CTGCTAAGAGCATCCGC	S–TGGGCAACAGAGGTTTTTCAAAAAG
		R–TGTTGGTGTGATGCACAG	A–CAATAACAACCTAATGGGCACTTCCTAATGCCAGA
−1577 C/T	rs11571316	F–CTGCTAAGAGCATCCGC	S–CTGGGGCTTGAAGGTTTCTATAATGTGTA
		R–TGTTGGTGTGATGCACAG	A–CAGTGTATAGAAAACAGGCAGGTCAGAAAAGGC
−1147 C/T	rs16840252	F_AGGGAGGCATTTGGTGA	S_GGACGGACTGGAGTAGGCAA
		R_CCGGAATTCTGTGCACTT	A_GTCATATTCCCTGTTACAACTGTCTGTTTGCATGT
−443 A/T	rs231774	F_AGAAGGATGGTGCTTCACA	S_GCCTAGTAGTTTTGGAGATGTCAATGAAATGA
		R_TGGAGAATTTCCTGGAGTACA	A_TGGACTGGATGGTTAAGGATGCCCAGAAG
+49 G/A	rs231775	F–TGCCTTGGATTTCAGCG	S–CCAGGTCCTGGCAGCC
		R–TGAAACAAATGAAACCCAGGTAG	A–AGGGATGAAGAGAAGAAAAAACAGGAGAGTGCAG
+923 C/T	rs231777	F_TGTGGGTTTCAGATGCAG	S_TGGCTAAGAAACCATGTAGTTTGTATGAAGTAG
		R_CTTTCCAGTATTGGGAGGG	A_CCATTGAATCTCAACCTTATCTCTCTCTAGACCTT
+6230 G/A	rs3087243	F–TTCATTCAGTATCTGGTGGAGTCTC	S–GGGATATAACATGGGTTAACACAG
		R–AAGGGGAGGTGAAGAACCTG	A–CATAGCAGTCCTTTATAAATCAATTGGCA

1 Performed by dynamic allele specific hybridisation with fluorescence resonance energy transfer (FRET) in a LightTyper©.

2 F, forward primer; R, reverse primer.

3 S, sensor; A, anch.

### Statistical Analysis

The STATA 9 software (Stata Corporation, College Station, TX, USA) was used to test for Hardy Weinberg equilibria (HWE) and odds ratios (OR) of genotype frequencies. Multivariate logistic regression analyses were applied to adjust for age, gender and ethnicities when comparing TB cases with all controls, PPD-negative and PPD-positive controls. Multivariate ordinal logistic regression analyses were performed to determine the influences of genetic variants on the severity scores of radiological signs. Calculations included adjustments for age, gender, ethnicity, recruitment centers and duration of cough, the latter because it was found correlated with the radiographic signs. Haplotype frequencies were estimated and compared using the haplo.score module of the haplo.stats package (http://cran.r-project.org/web/packages/haplo.stats/index.html).

Associations of the distinct disease phenotypes of opacities and cavities with *CTLA4* diplotypes comprising the +6230A allele were determined by weighted ordinal logistic regression analyses. Weights in the regression models were the posterior probabilities of each haplotype pair (diplotype) for each individual as estimated by the haplo.score module of the haplo.stats package.

Corrections for multiple testing of single variants were made by multiplication of P values with 7, the number of genetic variants that are not in strong linkage disequilibria with each other, and the two disease phenotypes examined. The final correction factor was, thus, 14 and corrected P values are given as P_cor_. To control haplotype analyses for multiple testing, 20,000 permutations were performed for global and haplotype-specific P values. The study sample provided 80% power to identify significant genotypic differences between TB cases and controls at a statistical support level of α = 10^−4^ with an OR of 1.4 and a minor allele frequency of 0.1 applying a multiplicative model.

## Results

The study group consisted of 2010 patients with pulmonary TB and 2346 controls group-matched for age, gender and ethnicity. Controls comprised 2219 purified protein derivative (PPD)-positive and 127 PPD-negative individuals

Genotyping of 8 SNPs located in the *CTLA4* gene showed allele distributions in HWE in both the patient and control groups. The minor allele frequencies (MAF) of variants was 15–46%, whereby the +6230 (ct60) A and G alleles were found in 19% and 81%, respectively, of participants. *CTLA4* −1722 showed a minor deviation from HWE in the control group but as this occurred in one subgroup only it was considered fortuitous (Table 3).

**Table 3:** Frequencies and distributions of SNPs studied^1^.

Genetic Variant	MAF^2^	Cases	Controls
		P^3^	P^3^
*CTLA4* −1765	0.46	0.54	0.40
*CTLA4* −1722	0.15	0.81	0.04
*CTLA4* −1661	0.16	0.49	0.51
*CTLA4* −1577	0.19	0.82	0.47
*CTLA4* −1147	0.16	0.82	0.57
*CTLA4* −443	0.17	0.73	0.53
*CTLA4* +49	0.38	0.62	0.65
*CTLA4* +923	0.25	0.73	0.09
*CTLA4* +6230	0.19	0.99	0.64

1 The χ2 test was applied to test for deviations from Hardy-Weinberg equilibrium.

2 Minor allele frequency.

3 P value for a deviation from Hardy-Weinberg equilibrium.

**Figure 1:**
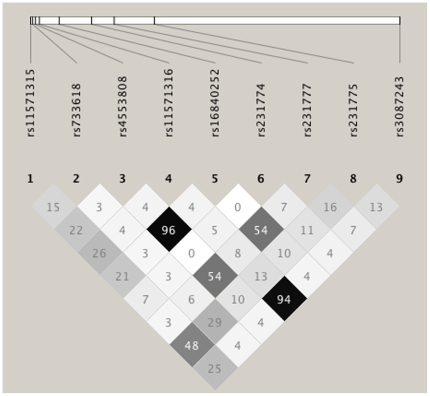
Pairwise linkage disequilibrium (r^2^) based on 9 *CTLA4* SNPs using HaploView 4.0 software. The figure depicts the strong LD of variants −1577/+6230 and of variants −1147/−1661. The positions relative to the ATG start codon of SNPs are given in Table 2.

The analysis of pairwise linkage disequilibrium (LD) revealed strong LDs between the variants −1577 with +6230 (R^2^ = 0.94) and −1147 with −1661 (R^2^ = 0.96) (Figure 1). Due to these LDs, the SNPs at positions −1577 and −1147 could safely be excluded from further analyses as these variants would not add additional information.

When comparing cases with controls, genotype frequencies were similar in all groups (Table 4) including subgroups of PPD-positive and PPD-negative controls (data not shown). Associations with disease severity were assessed by multivariate ordinal logistic regression based on quantitative estimates of radiographic signs of opacities and cavities. Age, gender, ethnicity, duration of illness, mycobacterial species (*M. tuberculosis* 69.1%, *M. africanum* 30.3%, *M. bovis* 0.6%), and recruitment site were included as potentially relevant co-variates. The stratifications showed that the A/A genotype of +6230 showed a significant association with low scores for opacities (Table 5) and a similar trend for cavities (data not shown). Compared to the most frequent +6230 G/G genotype, the A/A and A/G genotypes had odds ratios (ORs) of 0.39 (AA vs GG, confidence interval [CI] 0.23–0.65, P_cor_ = 0.005) and 1.06 (AG vs GG, CI 0.85–1.33, ns), respectively. As no difference was found between A/G and G/G and both genotypes showed similar ORs compared to A/A (GG vs AA, OR 2.57, CI 1.53–4.32, P_cor_ = 0.005 and AG vs AA, OR 2.72, CI 1.60–4.66, P_cor_ = 0.003), the disease-aggravating influence associated with the G allele appears to be dominant and the disease-mitigating effect associated with A recessive.

**Table 4:** *CTLA4* genotype frequencies in TB cases and controls^1^.

*CTLA4* SNP		Cases	Controls	OR^3^	95%CI^4^	p
	N	1990	2337			
−1765	AA	29.1%	29.1%	1		
	AG	49.0%	50.5%	0.97	0.8–1.1	0.623
	GG	21.8%	20.4%	1.04	0.9–1.2	0.639
	N	1986	2315			
−1722	AA	72.9%	70.2%	1		
	AG	24.9%	27.9%	0.88	0.8–1.0	0.067
	GG	2.2%	1.9%	1.18	0.8–1.8	0.435
	N	1952	2269			
−1661	AA	71.6%	70.6%	1		
	AG	25.8%	26.6%	0.94	0.8–1.1	0.364
	GG	2.6%	2.8%	0.89	0.6–1.3	0.552
	N	934	1057			
−443	AA	68.8%	68.0%	1		
	AT	28.1%	28.6%	0.98	0.8–1.2	0.852
	TT	3.1%	3.4%	0.90	0.5–1.5	0.680
	N	1912	2278			
+49	GG	40.1%	38.7%	1		
	GA	46.0%	47.4%	0.95	0.8–1.1	0.456
	AA	13.9%	13.9%	0.98	0.8–1.2	0.826
	N	903	1026			
+923	CC	54.7%	57.7%	1		
	CT	38.2%	35.2%	1.15	1.0–1.4	0.146
	TT	7.1%	7.1%	1.07	0.7–1.5	0.724
	N	1994	2328			
+6230	GG	64.6%	65.7%	1		
	GA	31.5%	30.5%	1.05	0.9–1.2	0.494
	AA	3.9%	3.8%	1.04	0.8–1.4	0.822

1 Analysed by logistic regression.

2 Variations due to assay failures.

3 Odds ratio.

4 Confidence interval.

**Table 5:** *CTLA4* genotype frequencies in TB cases stratified for the sizes of opacities in chest radiographs^1^.

	Opacity Score							
*CTLA4* SNP	0	1	2	3	OR^2^	95%CI^3^	P	P_cor_ 4
N	17	349	1075	364				
−1765 AA	0.0%	30.1%	29.6%	26.4%	1			
AG	64.7%	44.7%	49.3%	51.4%	1.09	0.9–1.4	0.498	
GG	35.3%	25.2%	21.1%	22.2%	0.85	0.6–1.1	0.268	
N	17	347	1074	363				
−1722 AA	82.3%	74.3%	72.6%	71.1%	1			
AG	17.6%	23.1%	24.9%	27.3%	1.14	0.9–1.5	0.277	
GG	0.0%	2.6%	2.4%	1.6%	1.09	0.5–2.3	0.822	
N	17	342	1056	357				
−1661 AA	52.9%	73.1%	71.9%	68.9%	1			
AG	47.1%	24.0%	25.9%	27.4%	1.05	0.8–1.3	0.692	
GG	0.0%	2.9%	2.3%	3.6%	1.25	0.6–2.4	0.505	
N	3	183	565	183				
−443 AA	100.0%	68.3%	69.2%	67.8%	1			
AT	0.0%	29.0%	27.1%	30.6%	1.03	0.8–1.4	0.838	
TT	0.0%	2.7%	3.7%	1.6%	0.83	0.4–1.7	0.618	
N	17	338	1040	347				
+49 GG	52.9%	45.9%	37.7%	40.6%	1			
GA	47.1%	42.6%	47.9%	46.1%	1.17	0.9–1.5	0.180	
GG	0.0%	11.5%	14.4%	13.3%	1.38	1.0–1.9	0.061	
N	3	180	545	175				
+923 CC	66.7%	51.7%	55.2%	56.0%	1			
CT	33.3%	41.1%	38.6%	34.3%	0.84	0.7–2.3	0.289	
TT	0.0%	7.2%	6.2%	9.7%	1.33	0.6–1.1	0.199	
N	17	350	1079	364				
+6230 GG	47.1%	63.1%	65.6%	63.2%	1			
GA	47.1%	29.4%	30.8%	35.2%	1.06	0.8–1.3	0.602	
AA	5.9%	7.4%	3.6%	1.6%	0.39	0.2–0.7	0.0004	0.005

1 Analysed by ordinal logistic regression.

2 Odds ratio.

3 Confidence interval.

4 Bonferroni-corrected by the factor of 14 for testing 7 SNPs not in strong linkage disequilibria and two disease phenotypes.

The haplotype −1765G/−1722A/−1661A/−443A/+49 G/+923C/+6230A was strongly associated with high opacity scores in a recessive model (global P_cor_ = 0.03; haplotype specific P_cor_ = 0.0005) and showed the same, albeit weaker, association with cavities (global P_cor_ = 0.03; haplotype specific P_cor_ = 0.03) (Table 6). The same applied to the diplotype containing homozygously this haplotype (opacities: haplotype specific P = 0.0005; cavities: haplotype specific P = 0.05; Table 7).

**Table 6:** *CTLA4* haplotype frequencies in TB cases stratified for the sizes of opacities and cavities in chest radiographs^1^.

	*CTLA4* SNP										
Haplotypes	−1765	−1722	−1661	−443	+49	+923	+6230	Freq.^4^	Score statistic^5^	P^6^	P_cor_ 7
Opacities	Global p = 0.02^2^, Global p_cor_ = 0.03^3^										
	G	A	A	A	G	C	A	0.19	−3.70	0.00021	0.00045
	A	A	A	T	G	C	G	0.08	−1.05	0.29	0.30
	A	G	A	A	A	C	G	0.15	−0.60	0.55	0.55
	A	A	A	T	G	T	G	0.06	−0.29	0.77	0.77
	G	A	G	A	G	T	G	0.13	0.39	0.69	0.70
	A	A	A	A	A	C	G	0.21	0.66	0.51	0.52
	G	A	A	A	G	C	G	0.11	0.68	0.50	0.50
Cavities	Global p = 0.005^2^, Global p_cor_ = 0.008^3^										
	A	A	A	T	G	C	G	0.08	−2.19	0.03	0.03
	G	A	A	A	G	C	A	0.19	−2.11	0.04	0.03
	G	A	G	A	G	T	G	0.13	−1.6	0.11	0.11
	G	A	A	A	G	C	G	0.11	−1.61	0.11	0.11
	A	G	A	A	A	C	G	0.15	−1.05	0.29	0.29
	A	A	A	T	G	T	G	0.06	0.15	0.88	0.88
	A	A	A	A	A	C	G	0.21	2.08	0.04	0.04

1 Seven tagging SNPs reported to appropriately indicate *CTLA4* haplotypes in sub-Saharan Africans (Ref. 13) analysed by the Haplo.score software assuming a recessive mode of inheritance.

2 P values obtained testing an overall association between haplotypes and the phenotype.

3 Global P values corrected for multiple testing by 20,000 permutations.

4 Frequency; only haplotypes with a frequency>0.01 are shown.

5 P values of haplotype-specific associations.

6 Simulated haplotype-specific P values corrected for multiple testing by 20,000 permutations.

7 Score statistic of the haplo.score software.

**Table 7:** *CTLA4* diplotype analysis in TB cases stratified for the sizes of opacities and cavities in chest radiographs.

Diplotype	Opacity Score						
	0	1	2	3	OR^1^	95% CI^2^	P^5^
N	17	305	961	331			
Other^3^/other	47.1%	60.3%	62.8%	61.3%	1		
HAPct60^4^/other	47.0%	31.8%	33.2%	36.9%	1.03	0.8–1.3	0.826
HAPct60/HAPct60	5.9%	7.9%	4.0%	1.8%	0.39	0.2–0.7	0.0005
	Cavity Score						
N	17	305	961	331			
Other/other	64.0%	60.9%	63.4%	60.4%	1		
HAPct60/other	31.0%	33.7%	33.2%	37.2%	1.03	0.8–1.3	0.77
HAPct60/HAPct60	5.0%	5.4%	3.4%	2.4%	0.59	0.3–1.0	0.05

1 Odds ratios (OR).

2 95% confidence intervals (CI) for the weighted ordinal logistic regression model with weights being the posterior probabilities for each diplotype for each individual.

3 Other, haplotype not comprising the +6230A allele.

4 HAPct60, haplotype comprising the +6230A allele (−1765 G/−1722 A/−1661 A/−443 A/+49 G/+923 C/+6230 A).

5 P values given for haplotype-specific associations.

## Discussion

Our findings do not provide evidence for a role of the +6230 polymorphism of human *CTLA4* in protection against pulmonary TB. On the other hand, our study design was sufficiently sensitive to detect a significant influence of the +6230 variant on the more detailed phenotypes of disease severity as radiograpically assessed by the extent of opacities and size and numbers of pulmonary cavities. The +6230 A/A genotype, which had been found negatively associated with autoimmune disease, was found to also be negatively associated with severe pathology in pulmonary TB. This applied to the major forms of pathology as identified by chest X ray. Thus, the *CTLA4* +6230 polymorphism was found to influence pulmonary TB only after *Mycobacterium tuberculosis* had overcome innate and adaptive host defence mechanisms.

Our data are partly supported by earlier studies in a murine model of TB. Using experimental aerosol infection of mice with *Mycobacterium bovis* bacillus Calmette-Guérin (BCG), it was shown that inhibition of CTLA-4 by antibody did not enhance protection [15]. Furthermore, in the same model, depletion of regulatory T cells, which express CTLA-4 constitutively, did not affect protection either [16]. However, the mouse model did not support a role of CTLA-4 in enhancing pathology. Although antibody mediated inhibition of CTLA-4 caused a rise in the number of T cells in lung-draining lymph nodes and an increase in the T-cell proliferative response to mycobacterial antigen, it did not result in increased lymphoid infiltration or granuloma formation in the lung assessed by careful histological examination [15]. As the radiological findings of opacities and cavities in human TB are considered to result from cellular infiltration and necrosis, respectively [17], our observations suggest that, in humans, a reduced activity of CTLA-4 causes an increased cellular infiltration of the lung, which may result from *in-situ* T-cell proliferation and recruitment of other cell types through cytokines. This is in agreement with the concept that pathology of TB to some extent is immune mediated, which has recently been confirmed by finding a clinical aggravation of pre-existing TB in the course of the immune reconstitution inflammatory syndrome (IRIS) seen among AIDS patients undergoing anti-retroviral therapy [18].

Collectively, the observation of the role of the *CTLA4* −6230 polymorphism and the occurrence of a haplotype/diplotype bearing the *CTLA4A* variant in pulmonary TB indicates that a reduced CTLA-4 activity in humans does not enhance protection against pulmonary TB but enhances pathology if containment of the infection fails. Accordingly, pulmonary TB appears not to belong to those infections which may contribute to explain the high prevalence of *CTLA4* +6230G in human populations. This evolutionary role may rather be with viral infections including hepatitis B, which was found to be controlled more effectively by carriers of the *CTLA4* +6230G allele than by those carrying the +6230A/A genotype [19]. In that study, the +6230G allele appeared to act in a dominant fashion, which is in agreement with the results obtained in autoimmunity [20] but at variance with our data on the severity of TB. An explanation has to await further functional studies.

The higher frequency of +6230G allele in the Ghanaian study group (>80%) compared to that found in Europeans (<60%) may suggest an evolutionary influence exerted by diseases that occur at higher prevalences in Africa than in Europe.

Our finding of strong LD in a study population from West Africa between variants at position +6230 and the SNP located in the promoter region of *CTLA4* (−1577) may open new avenues in the search for mechanisms which mediate the immunomodulatory effect of the +6230 variant.
